# Mediators of Periodontitis complementing the development of Neural Disorders

**DOI:** 10.12669/pjms.40.1.8097

**Published:** 2024

**Authors:** Ayesha Sadiqa, Munsara Khalid Khan

**Affiliations:** 1 Ayesha Sadiqa, BDS, M.Phil., Ph.D. Associate Professor, Department of Physiology, CMH Lahore Medical College and Institute of Dentistry, Lahore, Pakistan; 2 Munsara Khalid Khan, BDS. House Officer, Institute of Dentistry, CMH Lahore Medical College and Institute of Dentistry, Lahore, Pakistan

**Keywords:** Inflammation mediators, Somatosensory Disorder, Chronic periodontitis, Cytokines, and Chemokines

## Abstract

As a common oral health concern, periodontitis has been a source of attention for the global health community because of its linkage with systemic and neurological diseases. The purpose of the present study is to reveal the mediating role of specific cytokines, neuropeptides, and pathogens in the association of chronic periodontitis and neural disorders. To find the related literature different search engines namely PMC, Science Direct, PubMed, Research Gate, and Google Scholar were explored for a study period of five months from October 2022 to February 2023. This review offers a summary of those neuronal diseases that were more related to human behaviors in association with chronic periodontitis. Those neuronal pathologies mainly included Alzheimer’s disease, psychosis, stress, anxiety, dementia, Alzheimer’s, major depressive disorder, and diabetic peripheral neuropathy, which may otherwise remain subside or even control in the absence of chronic periodontitis and its mediators. Specifically, periodontitis related specific cytokines i.e. IL-6, IL-1, Tumor necrosis factor alpha (TNF-α), C-reactive protein (CRP), and alpha1-antichymotrypsin, neuropeptides such as insulin-like growth factor-2 (IGF-2), neuropeptide Y, substance P, neurokinin A, calcitonin gene-related peptide (CGRP), and vasoactive intestinal polypeptide (VIP), and a polybacterial pathogenic consortium of porphyromonas gingivalis, tannerella forsythia, and treponema denticola, were involved in the mediation and exacerbation of the associated neuronal cognitive pathologies.

## INTRODUCTION

Chronic periodontitis is a community oral disease that has been recognized as a leading cause of various systemic pathologies. It is a disease of continued inflammatory processes within the tooth periodontium including gingiva, alveolar bone, cementum, and periodontal ligaments (PDL) caused by debris and plaque growth, which carries colonies of bacteria over the tooth surface in the form of a biofilm.^1^,^2^

### Mediators of chronic periodontitis

Cytokines are the peptides that mediate in the cell to cell communication. Several cytokines including the acute phase reactants act as potential mediators in the progression of periodontitis in addition to causative pathogenic gram-negative bacteria.^3^ In the same regard, studies from Portugal and Pakistan have also confirmed the inter-relationship between circulatory Interleukin-6 (IL-6) and Chronic periodontitis, as elevated levels of serum IL-6, are seen in cases without periodontal therapy, and again reduced levels are observed in the treated studied population.^4^,^5^ Similarly, C-reactive protein (CRP) is also a known pro-inflammatory mediator of periodontitis as its plasma levels decline after treating chronic periodontitis through scaling and root planning.^6^

A novel Egyptian study was conducted on patients with chronic periodontitis to analyze the regulatory changes in the immune-inflammatory cytokines in relation to B-lymphocytes in them. The study revealed an increased levels of pro-inflammatory cytokines i.e. IL-6, interleukin-1 beta (IL-1β), and Tumor necrosis factor alpha (TNF-α), along with a significant rise of anti-inflammatory cytokines i.e. Transforming growth factor-beta (TGF-β), interleukin-35 (IL-35), and interleukin-10 (IL-10), in patients with stage-2 periodontitis, compared to controls. Moreover, the study also showed a raised population of B-lymphocytes in the same patients in comparison to the controls.^7^

Another study from Michigan declared that inflammatory cytokines namely IL-6, interleukin-8 (IL-8), and TNF-α are found to promote the degeneration of inflamed periodontal tissues. The quantification of the level of these inflammatory mediators in the gingival crevicular fluid (GCF) suggests that IL-6, interleukin-8 (IL-8), and TNF-α have been reported as relevant biomarkers in the pathophysiology of periodontitis.^8^

Nilsson et al., also explained in their review that the chemicals released by the causative bacteria activate the ongoing inflammatory process at the focal sites of periodontitis that further decays the soft and osseous tissues around the tooth. They concluded that an upsurge of pro-inflammatory interleukins i.e. interleukin-1 alpha (IL-1α), IL-1β, interleukin-12 (IL-12), and IL-6 along with TNF-α, found in periodontitis. Also, a pre-dominant expression was observed in such patients of other specific regulatory cytokines i.e. IL-1 receptor antagonist (IL-1Ra), interleukin-4 (IL-4), induced protein (IP-10), interferon- γ (IFN-γ), and IL-10. The study further elaborated on the excitatory role of IL-6, IL-1β, TNF-α, interleukin-17 (IL-17), Prostaglandin E-2 (PGE-2), and macrophage colony-stimulating factors in the osteoclastic deterioration of the bony socket around the tooth.^9^

### Steroids

A study from Cairo, Egypt supports the notion that certain hormones do play a vital role in the pathogenesis and deterioration of periodontitis. More than that the endocrine role of hormones like corticosteroids, androgens, estrogens, and progesterone has also proved a key factor in the formation of gingival pocketing through multiple channels such as clampdown immunity, aggravating exudation, osseous resorption progression and enhancing the fibroblastic action, terminating into clinical attachment loss and presenting periodontal pathology.^10^ Mohammed LJ et al. reported a high prevalence of 21% of periodontal pathologies in women who were using oral contraceptives compared to only 9% prevalence in the controls who were not using these varieties of steroids.^11^

### Neuropeptides

Certain neuropeptides play a key role in the potentiation of chronic periodontitis. A study supports the notion that there is a stimulatory role of gingival crevicular neuropeptide-Y (NPY) in the pathogenesis of periodontitis.^12^,^13^

A Japanese study explained that the nervous system plays a tremendous role in neurogenic inflammation through its neuromodulators mainly the neuropeptides. The study documented that certain neuropeptides including substance P (SP), NPY, vasoactive intestinal polypeptide (VIP), and more pronouncedly the calcitonin gene-related peptide (CGRP) were positively involved in bone metabolism and periodontitis.^14^

Sun C et al., tried to explore any association between periodontitis and major depressive disorder (MDD) at the gene level based on neuropeptides. They reported that insulin-like growth factor-2 (IGF-2) was highly expressed among both of the comparative diseases i.e. periodontitis and MDD.^15^ Recently in 2023, a thorough systematic review verified the direct association of NPY, SP, neurokinin A, CGRP, and VIP, with periodontitis.^16^ The aim of the current review was to reveal the mediating role of specific pro or anti-inflammatory cytokines, chemokines neuropeptides, and pathogens in the association of chronic periodontitis and somatosensory disorders.

## METHODS

It was a review of literature conducted to recognize the related published relevant content, retrieved through various search engines or professional databases such as Science Direct, PubMed Central, PubMed, Science Direct, Research-Gate, Google Scholar, and MEDLINE. To refine the search for relevant literature, the prime Mesh terms included Inflammation mediators, Somatosensory Disorder, Chronic periodontitis, Cytokines, Chemokines, Alzheimer’s Disease, Stress Disorders, Anxiety, diabetic neuropathies, and Schizophrenia.

The latest manuscripts published at least in the year 2009 were included and those published before 2009 were excluded from the review. All categories of manuscripts such as original articles, review articles, systematic review articles, meta-analysis reports, and case studies were included in the review as referenced articles. The manuscripts published in other than English language were also excluded.

Initially, more than a hundred related potential manuscripts were retrieved, which were then further screened by an in-depth reading of their titles, abstracts, and more specifically the conclusion of the manuscripts. Finally, only thirty-six manuscripts were selected to include as references in the current review article.

## RESULTS

### Chronic Periodontitis in Connotation with Nervous Disorders

Initiation of associated pathologies in the brain and other tissues is either through cytokine activation or even through direct bacterial invasion, which is the primary cause of periodontitis. It is suggested that periodontitis is positively linked with a systemic host response and with a low-grade inflammatory state, as assessed by raised serum levels of CRP and endothelial dysfunction.^17^

### Alzheimer’s disease and chronic periodontitis

**Fig.1:**
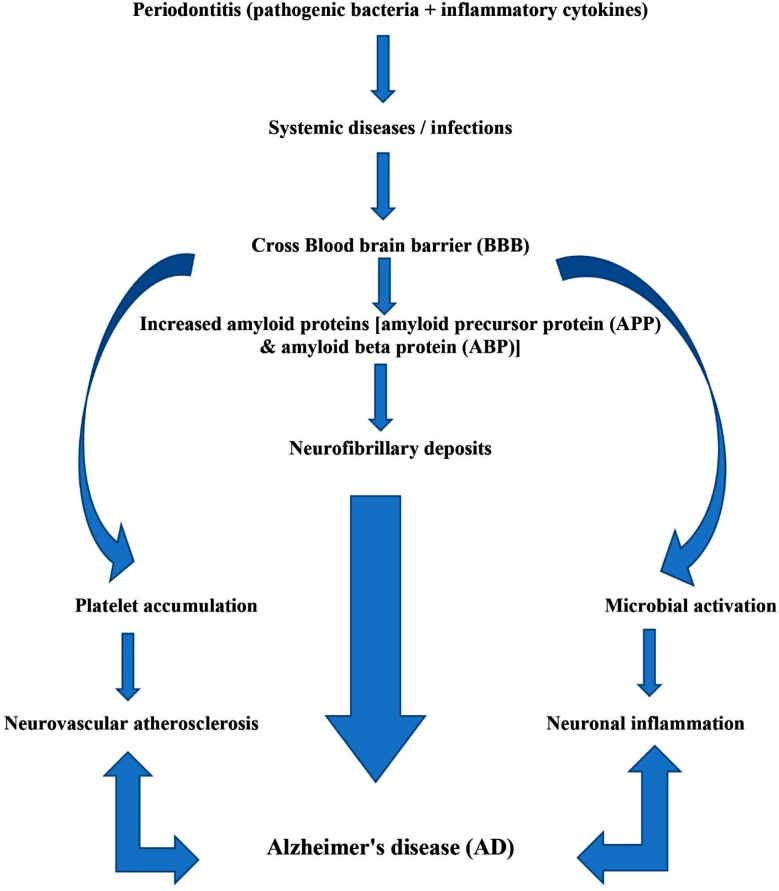
Flow chart showing mechanisms by which chronic periodontitis leads to Alzheimer’s disease.

Alzheimer’s disease (AD) is characterized by loss of memory and cognitive ability, which may lead to dementia. Neuro-inflammation is the underlying pathophysiology of AD which is correlated in terms of oxidative decay and low-grade inflammation with chronic periodontitis.^18^,^19^ Similarly, another study also declared that chronic periodontitis might be a predominant risk factor in the development of dementia.^20^ (Fig.1)

### Pathogens and their Pathophysiological mechanisms

Periodontitis acts as a possible risk factor by its microbial pathogenic spread to the neurological pathways and ultimately to the late onset of AD.^21^ The bacterial pathogens porphyromonas gingivalis, tannerella forsythia, and treponema denticola are the renowned bacteria involved in chronic periodontitis which on one hand act as a source of continuous irritant in the plasma to escalate inflammatory processes and on the other hand disseminate into the brain, due to close anatomical location.^22^

## DISCUSSION

The relationship between periodontitis and impaired memory is more pertinent in individuals above 60 years of age, as an infectious agent porphyromonas gingivalis is a particularly important serum pathogen, in the association of periodontitis with decreased cognitive abilities.^23^ A moderate persistent inflammatory progression was found in periodontitis due to causative pathogens that have a direct relationship with raised β-amyloid in advanced age. If this process kept on progressing, it would ultimately derail the cognitive abilities (dementia) and even turn into AD. In the same connection, a highly prevalent bacteria named porphyromonas gingivalis (key pathogen of periodontitis) is found to be significantly associated with the progression of AD, though the exact mechanism is still unclear.^24^

### Pathophysiological description

Periodontal disease has a potentiating effect on AD. The mechanism of this pathogenic linkage (initiating from periodontitis and leading to AD), is mainly through gram-negative bacteria and viruses which together enhance the pertinent cytokines, especially the ones that are considered to be the acute phase reactants (CRP). These organisms and their active mediators take cerebral entry and there they increase the concentration of amyloid precursor protein and amyloid beta protein which accumulates in the form of neurofibrillary deposits, causing cerebrovascular atherosclerosis and thus AD.^25^ (Fig.1)

### Role of cytokines

It was confirmed that alpha1-antichymotrypsin, IL-6, and to a smaller degree, C-RP are potentially involved in vascular brain diseases including AD and dementia.^26^ AD is a degenerative inflammatory pathology of neuronal cells mainly microglia, is considered a disease of old age. The most important basis of the strong connection between periodontitis and AD is inflammation which is expressed in terms of pro-inflammatory mediators mainly the CRP.^27^

Deposition of amyloid activates neuro-inflammation which causes cellular decay and reduces cognitive skills. The inflammatory mediators namely: IL-6, IL-1, and TNF- α, potentiate AD, and the same immune-inflammatory cytokines are also positively associated with chronic periodontitis and its obvious correlation with AD.^28^ (Table-I)

**Table-I:** Overview of common mediators of periodontitis and neural disorders.

Immune-inflammatory mediators	Category	Secretory cells	Neural disease along with chronic periodontitis	Possible role in the pathogenesis	References
C-reactive protein (CRP)	A pentameric plasma protein is released in response to acute-phase inflammatory reactions	Primarily produced by the hepatocytes, and to some extent secreted by vascular smooth muscle fibers, endothelial cells, & macrophages	Schizophrenia, Diabetic neuropathy, AD, and dementia	Alteration of oral microbiota, Progression of inflammation, and causing vascular endothelial dysfunction in brain tissues	6, 17, 26, 27, 35, 36, 37
IL-1α, IL-1β	Called ‘alarm cytokines, which are pro-inflammatory in nature	Mainly secreted by cells involved in innate immune e.g., granulocytes, macrophages, dendritic cells, and mast cells	Schizophrenia, AD	Potentiating neuro-inflammatory responses to amyloid deposits	1, 9, 28, 35
IL-4	Recognized as a prototypical immunoregulatory cytokine	Secreted by type-2 helper T-lymphocytes	Diabetic neuropathy, Mild Cognitive Impairment, and AD	Enhanced allergic responses mediated by IL-4 affect the expression of involved proteins of AD as well as brain inflammatory cytokine	9, 36, 37
IL-6	Pro-inflammatory cytokine	In response to infectious pathogenic bacteria, it is released by macrophages	Schizophrenia, Vascular brain diseases (AD, dementia)	Through progression of acute phase responses in immune-inflammatory reactions	4-5, 7-9, 26, 28, 35
IL-8, IL-9	A leukocyte chemoattractant	leukocytes, fibroblasts, and endothelial cells	AD and psychiatric illness (Schizophrenia)	It elevates the chronic neuroinflammation of AD which may cause cerebrovascular damage	8, 35
IL-10	An anti-inflammatory cytokine	Secreted by type-2 helper T-lymphocytes	Neurodegenerative diseases: Multiple Sclerosis, AD, and Parkinson’s Disease	Immuno-suppressive role in the anti-inflammatory modulation of glial cell activation and preventing neuronal inflammation	7, 9
IL-35	A cytokine with anti-inflammatory properties	Secreted by suppressor (regulatory) T-lymphocytes	Diabetes, SLE, and CNS autoimmune diseases	By suppressing CNS autoimmunity by exciting anti-inflammatory cytokines	7
IL-1Ra	Interleukin-1 receptor antagonist, which is involved in host defense mechanisms, especially against endotoxin-caused damage	Mainly produced by macrophages	AD, Multiple Sclerosis (MS), Down’s Syndrome	Through exaggerated immune response in CNS-related neuroinflammatory disorders	9, 28
IP-10	It is a chemoattractant of pro-inflammatory lymphocyte type T-helper and type cytotoxic T cells	In response to IFN-γ and exotoxins, secreted by local endothelial cells, monocytes, and fibroblasts	Involved in multiple CNS diseases e.g. Parkinsonism, AD, and dementia	Involved in chemoattraction and N-terminal proteolytic alteration	9
IFN-γ	A vital cytokine of immunological reactions	Secreted by natural killer cells and activated T-lymphocytes	Diabetic neuropathy, Stroke, Cerebral traumata, and multiple sclerosis	Complex unclear role, as it is involved in protection (physiological) as well as pro-inflammation (pathological) in autoimmune neuroinflammation	9, 36, 37
Tumor necrosis factor alpha (TNF-α)	A major regulatory cytokine of immune-inflammatory reactions	Macrophages and helper type T-lymphocytes	Schizophrenia, AD, Multiple Sclerosis, and Parkinsonism	By increasing the production and decreasing the clearance of amyloid beta	7, 8, 9, 35
Transforming growth factor-beta (TGF-β)	Belongs to the family of growth factors that regulate many cellular reactions	Macrophages and helper type T-lymphocytes	Mainly the dementia	It depressed the clearance rate of amyloid beta and hence elevate the cognitive decline	7
PGE-2	A potent inflammatory mediator of several immunological events in the body	Cells of inflammation as well as fibroblasts	Ischemic brain injury, Parkinsonism, Schizophrenia, and AD	It has complex pleiotropic effects, one is the neuroinflammatory progression	9, 35
Neuropeptide-Y	An orexigenic peptide of the brain	GABAergic neurons	Anxiety, epilepsy, AD, and Parkinsonism	Modulate neurogenesis and decline the toxic effects of amyloid beta	12, 13, 14, 16
Neurokinin A	A neurologically active endogenous peptide (a tachykinin)	submucosal glands and goblet cells	Psychotic disorders, autism	Through stimulating immune responses	16
Substance P	A neurotransmitter of pain perception	Eosinophils, dendritic cells lymphocytes, and macrophages	Parkinsonism	It lowers cell membrane potential, contributing to the easy firing of neurons	14, 16
vasoactive intestinal polypeptide (VIP)	A neuropeptide that acts as a neuromodulator neurotransmitter	By the neurons of CNS and PNS	Anxiety, and depression	act as a neuroprotective transmitter involving neuronal differentiation by an activity-dependent neurotrophic factor	14
insulin-like growth factor-2 (IGF-2)	A growth-regulating protein hormone (neutral peptide)	Mainly by liver-cells	Associated with the memory enhancement processes	It helps to reverse AD by neuronal plasticity and related signal transduction	15
alpha1-antichymotrypsin	anti-inflammatory serine protease inhibitor	Mainly by hepatocytes, and some leukocytes	In declining peripheral neuropathy	By reducing MHCII activation in cells of inflammation	15, 26
Matrix metalloproteinases-2-1 (MMP-2-14), and Tissue inhibitors of metalloproteinases-2 (TIMP-2).	Proteinases	Extracellular matrix	Diabetes Mellitus Type 2, and diabetic neuropathy	Mainly through the re-regulation of microflora	36, 37
Porphyromonas gingivalis, Tannerella forsythia, and Treponema denticola	Gram-negative, anaerobic, pathogenic bacteria	Found in deep periodontal pockets	AD, Parkinsonism	Induce pro-inflammatory cytokine release and enhance neural inflammation	21-24, 27

***Conclusively*** it can be stated that periodontal pathology may lead to AD by three proposed routes: firstly, through the injurious effects of pathogens, secondly, through the indirect responses of those pathogens which enhance the systemic as well as neurological inflammation, and lastly the amyloid reaction in the cerebral vasculature.^29^

### Stress/anxiety in relation to chronic periodontitis

Stress/anxiety and periodontitis, are a two-way street, chronic periodontitis aggravates anxiety, and anxiety aggravates periodontitis. Research also confirmed the correlation between periodontitis and anxiety.^30^ The interrelationship exists between emotional or psychological stress and periodontitis because of immune reactions, firstly due to direct secretions of neurotransmitters and neuropeptides, and secondly by means of neuro-hormonal control mechanisms.^31^

Psychological factors are also considered a vital risk factor for periodontal pathology^32^ (stress exacerbates periodontitis).^30^ The cause of stress may be different in different scenarios, like it may be social, cultural, and spiritual, and also due to the demise of a spouse or children. Noticeably, job-related stress is certainly associated with periodontitis.^33^

Several other anxiety-related conditions may potentiate periodontitis such as in earthquake victims a potential link was revealed between insomnia and periodontitis.^34^ A psychological disorder like Schizophrenia has also been announced as a risk factor for periodontal diseases through common intervening mediators namely IL-1β, IL-6, IL-9, TNF-α, TNF-β, PGE-2, and CRP.^35^ (Table-I)

### Metabolic syndrome, Diabetic neuropathy, and Periodontitis

Evidence exists to propose the association of metabolic syndrome and periodontitis via altering periodontal microbiota, though scarcity prevails to confirm their positive association in both animal and human studies.^36^ The positive correlation between diabetes type-2 and periodontitis has been established so far in the literature through common mediators i.e. Matrix metalloproteinases-2-1 (MMP-2-14), IL-4, CRP, Interferon-gamma (IFN-γ), and Tissue inhibitors of metalloproteinases-2 (TIMP-2).^37^,^38^ (Table-I)

## CONCLUSION

On account of previous research, it can be concluded that chronic periodontitis is a contributor to certain neurological disorders including dementia, Alzheimer’s disease, and major depressive disorder through systemic mediators namely: IL-6, IL-1, TNF-α, CRP, alpha1-antichymotrypsin, IGF-2, neuropeptide Y, substance P, neurokinin A, calcitonin gene-related peptide, and vasoactive intestinal polypeptide, a pathogen consortium of porphyromonas gingivalis, tannerella forsythia, and treponema denticola bacteria.

### Authors Contribution:

**AS:** Did the conception and design of the study, final drafting, and approval of the article and she is responsible for the integrity and accuracy of this study.

**MKK:** Did the conception, drafting, and designing of the manuscript.
